# Understanding the Clinical Profile and Hospitalisation Patterns of Residents From Aged Care Facilities: A Regional Victorian Hospital Study

**DOI:** 10.7759/cureus.42694

**Published:** 2023-07-30

**Authors:** Ali Uthuman, Tae H Kim, Dinglin Gu

**Affiliations:** 1 Department of Rural Health, University of Melbourne, Shepparton, AUS; 2 General Medicine, Goulburn Valley Health, Sehpparton, AUS; 3 General Medicine, Goulburn Valley Health, Shepparton, AUS; 4 Internal Medicine, Western Health, Melbourne, AUS

**Keywords:** falls, victoria, australia, emergency medicine, residential aged care

## Abstract

Introduction

Residents of residential aged care facilities (RACFs) are typically frailer than their community-dwelling counterparts. They often present to the emergency department (ED) with varied health issues, frequently leading to hospital admissions. These admissions can exacerbate patient frailty and strain the healthcare system. Despite global efforts to reduce ED presentations from RACFs, effective strategies still need to be discovered. This study examines the clinical profile and hospitalisation patterns of RACF residents in a regional Victorian town.

Aims

The study aimed to assess the prevalence of ED presentations and representations from RACFs, investigate the causes and outcomes of hospital admissions stemming from these presentations, and evaluate the prevalence of documented (advanced care directives) ACDs within this patient cohort.

Methods

Following ethical approval, we conducted a retrospective analysis of 467 ED presentations from 310 RACF patients admitted to Goulburn Valley Health's (GVH) ED from January to June 2022. We collected and examined data on demographics, ACD existence, ED presentation characteristics, and hospital admissions, classifying admission reasons into eleven groups. Statistical analysis was performed with GraphPad Prism and IBM SPSS, using inferential tests and logistic regression to assess readmission odds at a significance threshold of p<0.05.

Results

Our study encompassed 310 patients from multiple RACFs, yielding 467 ED presentations. These constituted 2.28% of total ED visits and 9.85% of those aged 65 and above. Most of the cohort were females (59.4%), aged between 79 and 91. About 98 patients presented multiple times, and 48.2% of presentations led to hospital admissions, with 6.2% of admitted patients succumbing during the hospital stay. A documented ACD was absent in 42.9% of the cohort. Statistically significant results include a correlation between male sex and an increased frequency of ED representations (p=0.0422) and a longer ED stay duration for admitted patients (p<0.0001). No significant associations were found between ACD presence and ED representations, ACD and sex, or between age and duration of stay in the ED. Age did not differ significantly among patients with single or multiple presentations or between patients with or without ACD. Regarding fall-related presentations, no significant sex-based difference in admission rates was found. The duration of stay between surgical and medical admissions was also statistically indifferent.

Conclusion

Our study highlights the significant utilisation of ED services by RACF residents, mainly males. The substantial percentage of these presentations resulting in hospital admissions underlines the critical nature of these visits. The absence of ACD in a significant portion of the cohort and the lack of its influence on the frequency of representations signal the need for further exploration. The results underline the ongoing challenge of meeting the complex healthcare needs of RACF residents and emphasise the importance of gender-specific interventions and efficient hospital utilisation strategies to optimise healthcare delivery in this population. Future studies should further investigate the underlying reasons for these findings to inform targeted strategies for reducing unnecessary ED visits and hospital admissions. Furthermore, fall-related presentations necessitate comprehensive ED assessments and integrated management approaches.

## Introduction

Residents of residential aged care facilities (RACFs) are typically frailer than their community-dwelling counterparts. Studies have demonstrated that hospital admissions within this demographic are frequently associated with adverse health outcomes [1].

These individuals often present with many different illnesses in the emergency department (ED). Various studies have demonstrated slight deference in the most prevalent causes of ED presentations, but they are infections [2], cardiorespiratory illness, and musculoskeletal-related [3]. One study in Western Australia demonstrated that 25% of presentations were musculoskeletal, and 22% were fall-related [3]. These presentations frequently necessitate subsequent hospital admissions, adding to the burden on the healthcare system and potentially exacerbating the frailty of these individuals.

Healthcare providers and policymakers from Australia and worldwide have trialled various models to reduce ED presentations and hospital admissions from RACFs. These interventions have been met with varied degrees of success [4,5], and the search for effective strategies continues.

To contribute to this ongoing quest, the present study delves into the clinical profile and hospitalisation patterns of residents from various RACFs in a regional Victorian town. By exploring the specific circumstances in this regional context, we hope to garner insights that inform tailored healthcare strategies to improve the well-being of RACF residents and ease the strain on our healthcare system.

## Materials and methods

Ethical approval and study design

Approval from the Research Governance Unit of Goulburn Valley Health (GVH) was granted for the single-centre retrospective study at the GVH ED.

Participants, data collection and variables

Data were collected retrospectively for all patients admitted from RACF to the GVH ED from January 1, 2022, to June 30, 2022. Coding results identified 467 episodes of ED presentations from 310 patients within this timeframe, which were then manually interrogated to extract data related to the demographic profile (age, gender), the presence of an advanced care directive (ACD), ED presentation (duration of stay at ED, reason for admission, ED representation), and hospital admission (status of admission, duration of admission, patient outcome). The reason for admission was further classified based on the following categories: respiratory, fall-related, fracture-related, cardiovascular, infection, altered mental status, device-related, gastrointestinal, mental and behavioural, acute cerebrovascular, or other.

Statistical methods

The data were analysed using GraphPad Prism (GraphPad Software, Inc., La Jolla, CA) and IBM SPSS. Descriptive statistical analysis presents summary statistics as mean with standard deviation or median with interquartile range. Where appropriate, inferential statistical tests were used to identify differences between groups. The Chi-Square test and Fisher’s exact test were used to compare categorical data. The Mann-Whitney U test was used to compare categorical data to numerical data. Logistic regression analysis was used to investigate the odds of readmission between different variables. A p-value of <0.05 indicated statistical significance.

## Results

Our research included 310 distinct patients from multiple RACFs, leading to a total of 467 ED presentations. In the context of our study's timeframe, the overall number of ED presentations stood at 20,507. Furthermore, considering only those aged 65 and above, the ED presentations amounted to 4,677. Thus, presentations from RACFs accounted for roughly 2.28% of the total ED visits and 9.85% of ED presentations among those aged 65 and above, representing a modest yet significant proportion of ED utilisation.

The patient population was predominantly female, making up 59.4% (n=184) of the cohort, while males accounted for 40.6% (n=126). Their ages ranged from 53 to 107 years. However, the majority of individuals were aged between 79 and 91 years. The cohort's median age was 86 years, with a mean age slightly lower at 84.5 years (SD 9.078).

Regarding the ED representations, 212 patients presented once, while 98 patients experienced multiple presentations. Among the latter, a significant majority had two presentations (64.3%), followed by a smaller proportion with three (23.5%), four (5.1%), five (3.1%), six (3.1%), and a minimal number with seven (1.0%) representations.

Out of the 467 ED presentations, 242 (51.8%) did not result in hospital admissions. Conversely, 225 (48.2%) presentations led to hospital admissions. While 14 (6.2%) admitted patients passed away, 211 (93.8%) returned to the RACF.

Out of the total population, 42.9% (n=133) did not have a valid ACD documented in our hospital system. There is a significant relationship between sex and the frequency of ED representations (Chi-square test, p=0.0422). Male patients had more representation (38.1%) than females (27.2%). The common causes of ED presentations are listed in Table 1.

**Table 1:** Common reasons for the emergency department presentations *Percentage of admission vs. no admission among each reason for ED visits. ^#^Percetange of each reason among all reasons for ED visits.

Reasons	Admission (percentage*)	No admission (percentage*)	Total	Percentage^#^
Respiratory related	64 (79.01%)	17 (20.99%)	81	17.34%
Fall-related	44 (28.39%)	111 (71.61%)	155	33.19%
Fracture	9 (69.23%)	4 (30.77%)	13	2.78%
Cardiovascular related	15 (39.47%)	23 (60.53%)	38	8.14%
Infection	19 (65.52%)	10 (34.48%)	29	6.21%
Altered mental status	30 (65.22%)	16 (34.78%)	46	9.85%
Device related complications	3 (30%)	7 (70%)	10	2.14%
Digestive system disorders	14 (46.67%)	16 (53.33%)	30	6.42%
Mental and behavioural disorders	4 (50%)	4 (50%)	8	1.71%
Suspicion of acute stroke	12 (66.67%)	6 (33.33%)	18	3.85%
Other	11 (28.21%)	28 (71.79%)	39	8.35%

The Mann-Whitney U test found no significant difference in age between patients with a single ED presentation and those with multiple presentations (p=0.1606). The median age of patients in the single presentation group was 87.00 years, slightly higher than the median age of 85.00 years in the multiple representation group.

The Chi-square test explored the potential relationship between the presence of an ACD and the risk of ED representations. Despite slight differences in representation rates between patients with an ACD (35.03%) and without an ACD (27.07%), the test revealed no statistically significant association between ACD presence and representation (p=0.1357). It is important to note that, of the representation, 63.27% occurred among patients with an ACD, while the remaining 36.73% were among those without an ACD. Nonetheless, our data suggest that the presence or absence of an ACD did not significantly influence the likelihood of readmission.

The Chi-square test to see the potential association between ACD and sex revealed no significant association between these variables (p=0.3571). While ACDs were slightly higher in females (59.24%) than in males (53.97%), the difference was insignificant. Thus, our data suggest that sex does not significantly influence the likelihood of having an ACD in this study population.

Our Mann-Whitney U test analysis revealed no significant difference in age between patients with and without an ACD (p=0.6664). The median age was similar in both groups (86 years with ACD vs. 85 years without ACD). Therefore, the presence of an ACD did not appear to influence the age distribution in these ED significantly encounters.

In our logistic regression analysis (Table 2) investigating the predictors of representation, we found that only sex had a statistically significant impact (p=0.041), suggesting that being male modestly increased the likelihood of representation, a finding that echoes and confirms the results from our Chi-square test.

**Table 2:** Logistic regression analysis on representation to emergency department OD: odds ratio, ACD: advance care directive *p<0.05

Variable	OR	Standard error	p-value
Age	−0.003	0.003	0.383
Sex	0.110	0.054	0.041*
ACD	0.087	0.053	0.103

Utilising Spearman's rank correlation coefficient revealed a very weak, non-significant correlation between age and duration of stay in the ED (p=0.5267).

**Figure 1:**
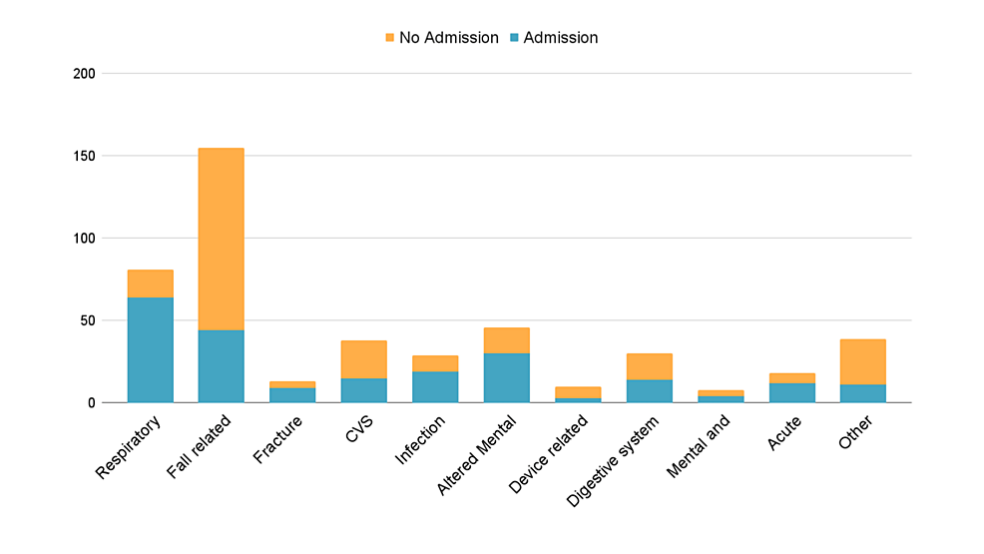
Common reasons for emergency department presentations

The Mann-Whitney U test revealed a significant difference in the duration of stay in the ED between patients who were admitted and those who were not (p<0.0001). Specifically, admitted patients had a median ED stay of 10.93 hours, compared to 6.40 hours for non-admitted patients. The ED presentation vs. admission status is given in Figure 1.

In our subgroup of patients who presented due to falls, 111 (71.61%) were not admitted to the hospital (61 females and 50 males), while 44 were admitted (31 females and 13 males). However, according to Fisher's exact test, this difference in admission rates between males and females within the fall patient group did not reach statistical significance (p=0.1023). Out of 155 fall-related presentations, 36 were discharged with a diagnosis of 'Fall', while 37 were discharged with a diagnosis of 'Mechanical Fall' (47%).

We compared the duration of stay between surgical and medical admissions using the Mann-Whitney U test. The median duration for medical admissions was 4.0 days (n=116), while surgical admissions had a median duration of 5.0 days (n=49). Despite the one-day difference in median duration, the analysis did not yield a statistically significant result (p=0.4861). The breakdown of admission units is given in Table 3.

**Table 3:** Breakdown of admission units at the Goulburn Valley Health

Unit	Numbers of admission
Critical care unit	1
Subacute unit (Mary Coram Unit - MCU)	5
Medical unit	116
Mental health unit	4
Respiratory unit	31
Short stay unit	19
Surgical unit	49

## Discussion

In our study, ED presentations from RACFs accounted for 2.28% of total presentations and 10% among individuals aged 65 and above. This figure is slightly higher than the 8.3% reported in a 2005 study conducted in Western Australia [3]. The increase in our study could reflect regional differences or potential changes in patterns of ED presentations from RACFs over time. Despite introducing innovative admission avoidance strategies, such as residential in-reach programmes, the proportion of ED presentations from RACFs in our setting still needs to be lowered. Further investigation into these factors is warranted.

In our study, females constituted the majority, accounting for 60% of the patient cohort. This proportion mirrors the gender distribution identified in a parallel study conducted in Western Australia, which also reported a similar predominance of female participants [3].

Our study identified a correlation between male sex and the incidence of ED representations. While there is a paucity of direct comparisons within the RACF context, research on the broader community-dwelling population provides some supporting evidence. For instance, a study conducted in North Florida found that males living in the community with multimorbid chronic diseases made statistically significantly more frequent ED visits [6]. Thus, the higher frequency of ED representations in males observed in our study may reflect a broader trend extending beyond the specific RACF context. Further research is warranted to better understand this association and its potential implications.

The rate of hospital admissions in our research approximated 50%, a statistic slightly below the findings of other similar studies. Crilly et al. observed a hospital admission rate of 76.6% among residents of RACFs [7]. Furthermore, research conducted in Queensland by Lukin et al. indicates that nearly half of the ED visits resulted in subsequent hospital admissions [8]. Lastly, an investigation by Finn et al. revealed an admission percentage of 60% [3]. The variations in these rates suggest regional or methodological differences across these studies.

Regarding the prevalence of ACDs, our study elucidated that 50% of the examined patients lacked a valid ACD within our hospital system. This finding contrasts with research conducted in rural Queensland's RACFs, where the existence of care preferences encompassing ACDs was remarkably high at 75% [9]. Alternatively, a 2015 investigation carried out in Metropolitan Melbourne revealed a notably lower prevalence, with ACDs existing among only 26% of RACF patients [10]. These disparities highlight the geographical variation in the uptake of ACDs and the need for further study into the factors influencing these patterns. It is important to highlight that a patient's ACD not being documented in the GVH computer system does not necessarily signify that the patient is without a valid ACD.

The study conducted by Finn et al. in Western Australia indicated that the average age at ED presentation was 83.7 [3]. This finding aligns closely with the data from our study, underscoring the consistency in patient demographics across different geographical locations.

While our research did not identify a statistically significant correlation between age and the duration of hospital stay in the ED, contrasting findings have been reported in other studies. Ogliari et al., for instance, identified a clear association between advanced age and extended stays within ED [11]. A similar trend was observed by Street et al., who found that being older, female, and a resident of a RACF was associated with a more extended hospital stay among elderly patients [12]. These divergent findings suggest that various factors may influence the relationship between age and length of hospital stay and warrant further investigation.

The PRO-AGE scoring system, a seven-variable model developed by Curiati et al., suggests that older individuals (above 90 years), males, or those who have had a recent hospital admission (within the last six months) exhibit an elevated risk of hospital admission and extended lengths of stay [13]. This predictive model highlights the multifaceted nature of risk factors contributing to hospital utilisation in the elderly population.

In our study, the increased length of stay at the ED due to hospital admission status can likely be attributed to patients awaiting bed availability in the suitable admitting unit. This finding points towards systemic issues related to hospital bed management and patient flow that may need to be addressed to improve ED efficiency and patient care outcomes.

A study by Owen et al. in the United States in 2006 revealed a higher incidence of falls among females [14]. Our research supports this finding, as we observed a similar trend in our study population, with 92 reported falls in females compared to 63 in males. This consistency points towards a possible gender-based disparity in the risk of falls, which merits further investigation.

Our study also reveals complexities in fall-related ED presentations among RACF residents. Of 155 cases, only 36 were discharged with a "Fall" diagnosis and 37 with a 'Mechanical Fall', which accounts for 47% of fall-related episodes. The remaining presentations culminated in various other diagnoses, suggesting that falls may often be symptomatic of, or exacerbate, other underlying health conditions. This divergence emphasises the importance of thorough geriatric assessments and holistic management strategies in the ED. It also highlights the need for targeted fall prevention interventions that account for the diverse health outcomes associated with falls in this population. It is also important to acknowledge that some patients with apparent clinical and/or radiological features of fractures at the time of ED presentation were categorised as 'fracture-related', even though a significant proportion, if not all, were due to falls.

Research conducted by Bo et al. in Italy indicates that the average length of stay for older hospitalised patients is approximately 11 days [15]. This finding provides insight into international comparisons of hospitalisation duration among this demographic.

Limitations

While our study provides valuable insights into the ED presentations of residents from multiple RACFs, several limitations warrant discussion. First, while substantial, our sample of 467 presentations was drawn from a single site over a six-month duration. This specificity may restrict the generalizability of our findings, as it might not account for broader variations or trends in different regions or timeframes. Second, the study's retrospective design necessitated reliance on existing medical records. Consequently, consistency in record-keeping, omissions, or incomplete patient histories could affect the reliability of our data and, in turn, the study's outcomes. Third, this study did not consider potential confounding variables such as variations in individual health statuses, the standards of different RACFs, or varying healthcare policies, all of which could influence ED presentations, hospital admissions, and ACD prevalence. Lastly, the study's cross-sectional design limited our ability to capture temporal changes or establish causal relationships. These constraints necessitate further longitudinal research to better understand the dynamics within this patient demographic. Future studies should address these limitations, thereby providing a more comprehensive and generalizable understanding of the ED presentations of residents from RACFs.

## Conclusions

Our research delineates patterns of ED presentation among RACF patients, accounting for approximately 10% of ED presentations among patients above 65 years during the study period. The data reveal a significant relationship between patient sex and ED representation frequency, with males showing higher rates. However, factors like age and the presence of an ACD did not impact the likelihood of ED representation. Admitted patients had longer ED stays than non-admitted ones, but patient age did not significantly correlate with the duration of stay in the ED. Despite surgical admissions having longer durations than medical ones, the difference was not statistically significant. Furthermore, our analysis of fall-related presentations underscores the complex nature of falls among RACF residents, indicating the necessity for comprehensive patient assessments, multifaceted management strategies, and targeted fall prevention interventions in this population. These combined findings provide critical insights for healthcare providers and policymakers aiming to enhance care delivery and planning for RACF patients.
